# The overlooked manipulation of nucleolar functions by plant pathogen effectors

**DOI:** 10.3389/fpls.2024.1445097

**Published:** 2024-08-07

**Authors:** Sarah Ranty-Roby, Frédéric Pontvianne, Michaël Quentin, Bruno Favery

**Affiliations:** ^1^ INRAE, Université Côte d’Azur, CNRS, Institut Sophia Agrobiotech (ISA), Sophia Antipolis F-06903, Sophia Antipolis, France; ^2^ CNRS, Laboratory of Plant Genome and Development, Perpignan, France; ^3^ International Research Organization for Advanced Science and Technology, Kumamoto University, Kumamoto, Japan

**Keywords:** effector, nucleolus, plant immunity, pathogens, nucleolar functions

## Abstract

Pathogens need to manipulate plant functions to facilitate the invasion of their hosts. They do this by secreting a cocktail of molecules called effectors. Studies of these molecules have mostly focused on the mechanisms underlying their recognition and the subsequent transcriptional reprogramming of cells, particularly in the case of R gene-dependent resistance. However, the roles of these effectors are complex, as they target all cell compartments and their plant targets remain largely uncharacterized. An understanding of the mechanisms involved would be a considerable asset for plant breeding. The nucleolus is the site of many key cellular functions, such as ribosome biogenesis, cellular stress regulation and many other functions that could be targets for pathogenicity. However, little attention has been paid to effectors targeting nucleolar functions. In this review, we aim to fill this gap by providing recent findings on pathogen effectors that target and manipulate nucleolar functions and dynamics to promote infection. In particular, we look at how some effectors hijack ribosome biogenesis, the modulation of transcription or alternative splicing, all key functions occurring at least partially in the nucleolus. By shedding light on the role of the plant nucleolus in pathogen interactions, this review highlights the importance of understanding nucleolar biology in the context of plant immunity and the mechanisms manipulated by plant pathogens.

## Introduction

Plant-pathogen interactions involve a complex molecular dialogue between the interacting partners. In particular, pathogens inject or secrete a cocktail of molecules, known as effectors, into the plant, to alter plant functions for the benefit of the pathogen or to suppress plant defenses. The term “effector” is not limited to interactions between plants and pathogenic micro-organisms. It also encompasses plant-pest interactions and microbes that are beneficial to the plant. Effectors were initially considered to be any secreted protein recognized by the host, but their definition now includes any molecule that alter host physiology and pathogenicity (Hogenhout et al., 2009). Effectors hijack various host-cell functions to facilitate colonization by the pathogen according to their developmental stage and feeding strategy. In particular, the nucleus and its functions are targeted by many effectors of importance for beneficial and parasitic interactions (Harris et al., 2023; Tehrani and Mitra, 2023).

**Figure 1:**
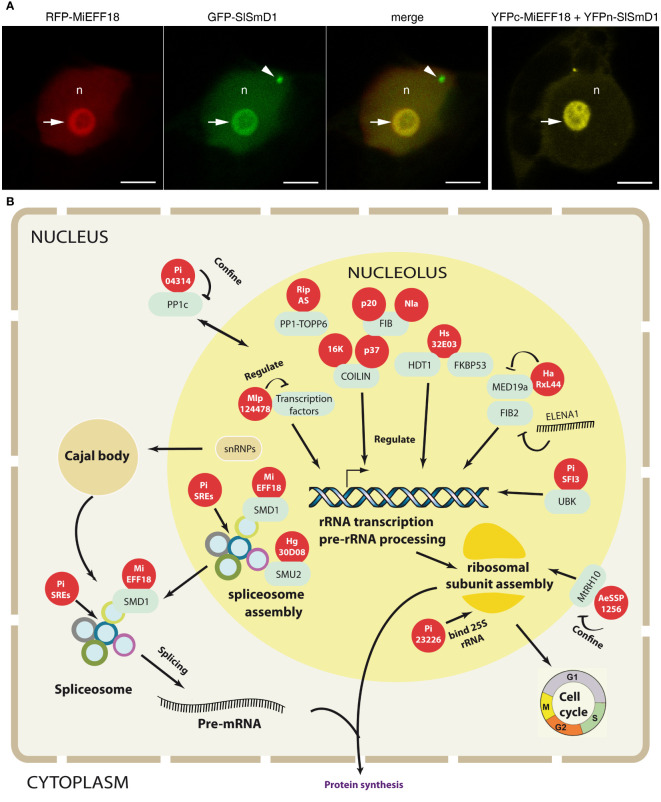
The nucleolus is targeted by plant pathogen effectors. **(A)** Root-knot nematode MiEFF18 effector interact with the plant spliceosomal protein SmD1 in the nucleolus. On the left (first three images), co-localization of RFP-MiEFF18 and GFP-SlSmD1 in *Nicotiana benthamiana* leaf epidermal cells. On the right, a bimolecular fluorescence complementation (BiFC) experiment showing interactions of YFPc-MiEFF18 with SlSmD1-YFPn. n, nucleus; arrow, nucleolus; arrowhead, nucleoplasmic speckle; Bars, 5 μm (Mejias et al., 2021). **(B)** Molecular mechanisms targeted by pathogen effectors in the nucleolus. Pathogen effectors (red circles) interact with various plant proteins (blue circles) and affect functions that occur partly or entirely within the nucleolus, modulating plant response. Effectors modulate gene expression, ribosome biogenesis and splicing activity by affecting spliceosome assembly, which occurs partly in the nucleolus. Finally, effectors can alter specific functions by modifying protein localization, preventing entry into the nucleolus (i.e., by confining the protein to a particular cell compartment).

The nucleus has a sophisticated dynamic internal structure, including numerous nuclear bodies associated with compartmentalized functions (Shaw and Brown, 2004; Muñoz-Díaz and Sáez-Vásquez, 2022). The nucleolus is the largest nuclear body (**Figure 1A**). It is a membrane-less structure present during interphase that disappears when the cell divides. The nucleolus has several different regions: fibrillar centers (FC; both heterogeneous and homogeneous in plants), dense fibrillar components (DFC), and granular components (GC) (Muñoz-Díaz and Sáez-Vásquez, 2022). These structures reflect the ribosome biosynthesis processus that occurs in a vectorial pattern within the nucleolus, starting with the transcription of pre-rRNA in the FC, early processing steps of pre-rRNA in the DFC, while the later processing and RNA modification steps, together with the formation of pre-ribosomal particles, occur in the GC (Boisvert et al., 2007). In some cases, plant nucleoli also contain nucleolar cavities, the role of which remains unknown (Pendle et al., 2005; Brown and Shaw, 2008). However, recent proteomics data have suggested additional roles for this subnuclear compartment (Pendle et al., 2005; Palm et al., 2016; Montacié et al., 2017), including involvement in cell-cycle regulation (Hernandez-Verdun, 2011; Zakrzewska-Placzek et al., 2023), in telomere maintenance and genome organization (Pontvianne et al., 2016; Bersaglieri and Santoro, 2019; Pontvianne and Liu, 2020). The nucleolus is also involved in the response to various stresses, such as DNA damage (Rubbi and Milner, 2003), and in protein sequestration (Wang et al., 2019a).

The nucleolus of *Arabidopsis thaliana* may contain up to 1602 proteins, together with (pre)-ribosomal rRNA and DNA (Palm et al., 2016). Fibrillarin (FIB), nucleolin and B23 are three major proteins essential for nucleolus function. FIB plays an important role in pre-rRNA maturation, through its methyltransferase activity, which directs the 2’-O-ribose methylation of pre-rRNA. Nucleolin and B23 are involved in rDNA transcription, pre-rRNA processing, rRNA maturation, and ribosome assembly and export (Pontvianne et al., 2007; Murano et al., 2008; Kalinina et al., 2018). A fourth protein, Coilin, present mostly in Cajal bodies — distinct subnuclear structures present in eukaryotic living cells and frequently associated with the nucleolus (**Figure 1B**) — ostensibly plays an important indirect role in certain nucleolar functions (Shaw and Brown, 2004; Muñoz-Díaz and Sáez-Vásquez, 2022). Cajal bodies are thought to play a major role in spliceosome activity, and most small RNAs present in the nucleolus are also present Cajal bodies (Morris, 2008).

Given its multiple functions, it is not surprising that the effectors of many pathogens are meant to hijack the nucleolus (Kalinina et al., 2018). In a recent review, Harris et al. (2023) reported 72 effectors that have been shown to target the host nucleus and for which specific host targets have been identified. However, studies focusing on the targets of plant pathogen effectors have largely overlooked the nucleolus. This review aims to fill this gap, focusing on this specific subcellular compartment, which has recently come to the fore as a prominent venue for plant pathogen effectors. The primary aim of this review is to provide a detailed description of pathogen nucleolar effectors and their plant targets, focusing particularly on the mechanisms by which these effectors manipulate plant functions in the nucleolus.

## Nucleolar effectors and their targets

The nucleolus has been identified as an important target for several pathogens in various human and animal hosts (Bierne, 2013; Iarovaia et al., 2021). In human cells, the first nucleolar effector to be described was the *E. coli* pathogen effector EspF, which is highly destructive, disrupting a subset of nucleolar factors through the relocalization of Nucleolin, an essential protein for nucleolus activity (Dean et al., 2010). Human pathogens can disrupt nucleolar functions, thereby also disrupting innate immune signaling. For example, *Coxiella burnetii* nucleolar effector A (NopA) binds to Ran GTPase and promotes the nuclear accumulation of Ran(GTP), thereby perturbing the import of the transcription factor NF-κB and innate immune signaling (Burette et al., 2020). Along similar lines, the *Brucella abortus* bacterial effectors NyxA and NyxB target SENP3 (sentrin-specific protease 3) to modulate the subcellular distribution of nucleolar proteins and enable pathogen replication (Louche et al., 2023). Given its importance for animal pathogens, it is expected that the nucleolus is also hijacked by plant pathogen and pest effectors (**Table 1**).

**Table 1:** Effectors targeting at plant nucleolus. Effectors from viruses (in blue), bacteria (in yellow), filamentous pathogens (in green) and nematodes (in grey) are targeting known or unknown nucleolus functions.

Effectors	Pathogen	Subcellular localization(s)	Biochemical properties	Target(s)	Function(s)	Reference
**p37**	*Pelargonium* line pattern virus	nucleolus, Cajal bodies	nd	Fibrillarin and Coilin	Impact on virus biological cycle	(Pérez-Cañamás et al., 2022)
**SatBaMV encoded P20**	Bamboo mosaic potex virus (BaMV)	nucleolus, nucleus	Non-structural protein	Fibrillarin	Regulation of the systemic trafficking of the virus by the Fibrillarin-satBaMV-P20 RNP complex	(Chang et al., 2016)
**Multifunctional nuclear inclusion protein (Nla)**	Potato virus A	nucleolus, nucleus	Vpg domain	Fibrillarin	Regulation of the infection cycle	(Rajamäki and Valkonen, 2009)
**16K**	Tobacco rattle virus	nucleolus	cysteine-rich silencing suppressor	Coilin	Induction of SA-responsive genes	(Shaw et al., 2019)
**ORF3**	Groundnut rosette virus	nucleolus, Cajal bodies	Arginine rich and leucine rich region and motif LXXLL	nd	Promotion of long-distance movement	(Kim et al., 2007)
**DspA/E**	*Erwinia amylovora*	nucleolus, cytoplasm	Avirulence protein	Unknown	Repression of *de novo* protein synthesis and cell death regulation	(Degrave et al., 2013)
**RipAS**	*Ralstonia solanacearum*	nucleolus	nd	Protein phosphatase (PP1) StTOPP6	Reduction of nucleolar accumulation of STOPP4 and promotion of bacterial wilt	(Wang et al., 2023)
**ELF18-INDUCED LONG NON CODING RNA 1 (ELENA1)**	*Pseudomonas syringae* pv *tomato*	nucleolus	Long non-coding RNA	Fibrillarin	Dissociation of the FIB2/MED19a complex and release of FIB2 from PR1 promoter to enhance PR1 expression	(Seo et al., 2019)
**AeSSP1256**	*Aphanomyces euteiches*	nucleolus	SP, NLS, small protein	MtRH10	Interaction with a functional nucleocytoplasmic RNA helicase and promotion of pathogen infection	(Camborde et al., 2022)
**Mlp124478**	*Melampsora larici-populina*	nucleolus	SP, NLS, DNA binding domain, belongs to protein family with positive selection	16 protein interactors	nd	(Ahmed et al., 2018)
**SFI3/Pi06087/PexRD16**	*Phytophthora infestans*	nucleolus, nucleus	RXLR effector	UBK	Suppression of early immune transcriptional responses	(He et al., 2019)
**PvAVH35, PvAVH133, PvAVH67, PvAVH36, PvAVH47, PvAVH135, PvAVH3**	*Plasmopara viticola*	nucleolus, nucleus	RXLR effectors	nd	Suppression of BAX-triggered cell death	(Chen et al., 2020)
**ATR13**	*Hyaloperonospora arabidopsidis*	nucleolus, nucleus, cytoplasm	RXLR effector with a disordered region	nd	nd	(Leonelli et al., 2011)
**HaRxL44**	*Hyaloperonospora arabidopsidis*	nucleolus, nucleus	RXLR effector	Mediator subunit 19a (MED19a),	Interaction with MED19a, shifting the balance of defense transcription from SA-responsive defense to JA/ET-signaling,	(Caillaud et al., 2013)
**PmEC01597, PmEC03792**	*Phyllachora maydis*	nucleolus, nucleus, plasma membrane	nd	nd	nd	(Helm et al., 2022)
**Pi04314**	*Phytophthora infestans*	nucleolus, nucleus	RXLR effector	Three host protein phosphatase 1 catalytic (PP1c) isoforms	Re-localization of the targets from the nucleolus to the nucleoplasm, promoting late blight disease.	(Boevink et al., 2016)
**SFI1, SFI2, PexRD24, PexRD20, PexRD25, PexRD49; PexRD16, CRE5, Avr4, AvrSmira 1**	*Phytophthora infestans*	nucleolus, nucleus	RXLR effectors	nd	Enhanced pathogen leaf colonization	(Wang et al., 2019b)
**ChEC74, ChEC98, ChEC104, ChEC108, ChEC111**	*Colletotrichum higginsianum*	nucleolus, nucleus	nd	nd	nd	(Robin et al., 2018)
**Pi23226**	*Phytophthora infestans*	nucleolus	nd	3′-end of 25S rRNA precursors	Nucleolar inflation, alteration of ribosome biogenesis: ribosome malfunction and cell death induction, beneficial for pathogenesis	(Lee et al., 2023)
**30D08**	*Heterodera glycines*	nucleolus, nucleus	SP, two NLS	SMU2 (homolog of suppressor of mec-8 and unc-52 2)	Alteration of pre-mRNA splicing for expression of genes important for feeding site formation	(Verma et al., 2018)
**MiEFF16**	*Meloidogyne incognita*	nucleolus, nucleus	SP, NLS	Unknown	nd	(Mejias et al., 2021)
**MiEFF18, MeEFF18**	*Meloidogyne incognita, Meloidogyne enterolobii*	nucleolus, nucleus	SP, NLS, NoLS	SmD1, an essential component of the spliceosome	Alteration of alternative splicing and proteome diversity, and of the formation of nematode-induced giant cells	(Mejias et al., 2021, 2022)
**32E03**	*Heterodera schachtii*	nucleolus	SP, NLS	FKBP53 (histone chaperone) and HDT1 (histone deacetylase)	Modification of rRNA gene expression leading to a higher number of ribosome and enhance nematode pathogenicity	(Vijayapalani et al., 2018)
**22E10, 13G11**	*Globodera pallida*	nucleolus, nucleus	SPRYSEC effector proteins	nd	nd	(Jones et al., 2009)

NLS, nuclear localization signal; NoLS, nucleolar localization signal; SP, signal peptide; nd, not determined.

The manipulation of nucleolar functions has been shown to be essential for viral virulence and replication, and has long been considered typical of, and exclusive to those pathogens (Taliansky et al., 2010). For example, the groundnut rosette virus open reading frame (ORF) 3 localizes in the nucleolus and Cajal bodies allowing long-distance transport of the virus (Kim et al., 2007). Potato virus A produces a multifunctional nuclear inclusion protein that localizes to the nucleus and nucleolus, disrupting FIB, which appears to be essential for viral infection in plants (Rajamäki and Valkonen, 2009). Another plant virus, bamboo mosaic virus (BamV), also targets FIB, which has been shown to be required for long-distance phloem transport (Chang et al., 2016). Recent studies have shown that the *Pelargonium* line pattern virus (PLPV) produces an effector called p37 that interacts with the two nucleolar proteins, FIB and COILIN, this interaction leading to restriction of the antiviral response (Pérez-Cañamás et al., 2022).

Plant bacterial pathogens have also been shown to target biological functions of the nucleolus. Interestingly, the pathogen-associated molecular pattern (PAMP) ELF18 from *Pseudomonas syringae* pv. *tomato* has been shown to induce ELENA (ELF18-INDUCED LONG NONCODING RNA 1), disrupting the FIB2/MED19a complex, to fine-tune the immune response (Seo et al., 2019). In this case, the effector targets FIB to activate plant defenses. By contrast, *Erwinia amylovora*, the causal agent of fire blight, produces an avirulence protein, DspA/E, which represses *de novo* protein synthesis and triggers cell death during its necrotrophic phase (Degrave et al., 2013). A type III effector from *Ralstonia solanacearum*, RipAS was recently reported to reduce the accumulation of protein phosphatase 1 (PP1) in the nucleolus, thereby promoting bacterial wilt (Wang et al., 2023). However, the characterization of bacterial nucleolar effector targets remains, even in human pathogens (Hanford et al., 2021), much less frequent than that of the effector targets of fungal pathogens or oomycetes.

Indeed, several such effectors have been described in filamentous fungal and oomycete pathogens (Caillaud et al., 2012; Petre et al., 2015; Pecrix et al., 2019). *Melampsora larici-populina*, the causal agent of poplar rust, produces the Mlp124478 effector, which binds to TGA1a binding site sequences in DNA to modulate gene expression (Ahmed et al., 2018). *Aphanomyces euteiches* produces a small protein known as AeSSP1256 that specifically interacts with a plant RNA helicase implicated in ribosome biogenesis. The AeSSP1256 effector causes enlargement of the nucleoli and ribosome-associated gene suppression, facilitating pathogen infection (Camborde et al., 2022). *Hyaloperonospora arabidopsidis* produces the HaRxL44 effector to degrade the mediator complex subunit MED19, shifting the balance of defense responses from salicylic acid (SA) responses to jasmonic acid (JA) and ethylene (ET) signaling (Caillaud et al., 2013). *Plasmopara viticola*, the causal agent of downy mildew, produces numerous RxLR effectors (i.e. proteins with an RxLR motif facilitating effector translocation within the plant cell) effectors that localize in the nucleolus and can suppress BAX-triggered cell death. Another major oomycete pathogen, *Phytophthora infestans*, the causal agent of potato late blight disease, has been shown to control ribosome biogenesis with the nucleolar effector Pi23226 (Lee et al., 2023). *P. infestans* also produces the Pi04314 RxLR effector, which targets three host PP1 catalytic subunit (PP1c) isoforms to promote late blight disease (Boevink et al., 2016). A number of other effectors of filamentous pathogens have also been shown to localize in the nucleolus (**Table 1**) although their precise targets have yet to be characterized.

Finally, other plant pests, including plant-parasitic nematodes in particular, also possess effectors that hijack the nucleolus (Mejias et al., 2019). The 30D08 effector of the cyst nematode *Heterodera glycines* (Verma et al., 2018) and the conserved root-knot nematode EFFECTOR 18 (MiEFF18 and MeEFF18) (Mejias et al., 2021, 2022) target auxiliary or core spliceosomal proteins. The 32E03 effector of the cyst nematode *Heterodera schachtii* targets a histone deacetylase (HDT1), thereby altering rRNA gene transcription, affecting ribosome biogenesis and promoting nematode pathogenicity (Vijayapalani et al., 2018). The cyst nematode *Globodera pallida* produces SPRYSEC effectors, which enter the nucleolus but the targets of which remain to be identified (Jones et al., 2009). The mechanisms underlying these examples are described in greater detail below.

## What mechanisms are targeted in the nucleolus?

Beyond the effector-target interaction, the mechanisms by which the pathogen manipulates nucleolar functions are of particular interest given the essential functions of this compartment. Ribosome production and composition, spliceosome formation, the regulation of gene expression and telomere maintenance have been shown to be key elements in plant growth and development, but our knowledge of the role of these nucleolar functions in plant pathogen responses remains essentially descriptive (Kalinina et al., 2018). However, several recent studies, including some functional studies, have shed light on how pathogens can manipulate these functions to their advantage (**Figure 1B**).

### Ribosome biogenesis as a target of pathogens

Ribosome biogenesis is one of the key functions of the nucleolus. Indeed, the nucleolus is formed as a consequence of ribosome biogenesis (Picart and Pontvianne, 2017). However, even though this process is essential for gene expression, it was only recently shown to be hijacked by pathogens. The Pi23226 effector has been shown to induce nucleolar inflation during the biotrophic/necrotrophic phase transition of *P. infestans* after the infection of tobacco plants and just before the induction of cell death. The oomycete then feeds on cell debris during its necrotrophic stage, increasing host susceptibility (Lee et al., 2023). Further analyses showed that the effector binds to the 3’ end of 25S rRNA precursors, preventing the further processing of 27S pre-rRNAs, with an impact on ribosome biogenesis at later stages. In consequence, newly produced ribosomes are functionally affected, leading to a global inhibition of protein translation. This work therefore strongly suggests that Pi23226 manipulates ribosome biogenesis to induce cell death (Lee et al., 2023). An *H. schachtii* effector, 32E03, also targets ribosome biogenesis by regulating rRNA gene expression at the chromatin level. This effector interacts with HDT1 regulating rRNA gene transcription. In the presence of this effector, rRNA epigenetic silencing is abolished and most rRNA genes are actively transcribed. Ribosomes are essential for protein production and this process is hijacked by the pathogen to regulate the number of rRNA molecules present to its own advantage (Vijayapalani et al., 2018). Ribosome biogenesis is also known to be closely linked to cell-cycle regulation (Boisvert et al., 2007; Derenzini et al., 2017), and cell-cycle perturbations are thought to trigger cell death (Yoshiyama, 2016). Thus, alterations to ribosome biogenesis are of great potential benefits to pathogens, to facilitate the production of proteins they need for pathogenicity or their own development, or to redirect the efforts of the plant away from its defenses, enabling the pathogen to evade these defenses. Further systematic research on effector proteins will undoubtedly reveal other examples.

### Manipulation of gene transcription

Pathogens can also modulate the transcription of genes in order to establish conditions in which they can thrive within their hosts. For example, HaRxL44, a downy mildew effector, strongly deregulates plant defense gene expression (Caillaud et al., 2013). This effector targets the mediator complex — more specifically the MED19a subunit — thereby strongly affecting the expression of plant defense genes. Its binding to MED19a leads to its degradation by the proteasome machinery. This results in the activation of JA/ET defense pathways and, thus, inhibition of the salicylic acid (SA) defense pathway, through the inhibition of *PR1* gene expression in particular (Caillaud et al., 2013). Similarly, upon PAMP detection, ELENA1 dissociates the FIB2/MED19a complex to release FIB2 and enhances *PR1* gene expression, resulting in a strong alteration of the immune response. The mediator complex, consisting of 25 proteins, is highly conserved in eukaryotes and mediates the interaction between transcriptional regulators and RNA polymerase II. It has been shown to be a target of choice for modulating host transcription, particularly in plant immune responses (Seo et al., 2019; Chen et al., 2022). The nucleolar effector of *M. larici-populina*, Mlp124478, binds directly to the DNA sequences corresponding to the TGA1a binding sites of multiple transcription factors modulating plant defense gene expression: *WRKY27*, *WRKY33*, *MYB51*, *NHL3*, *RPP8*, *YSL9*, *AZI1*, *RK11*, *JAZ1*, *ASA1* and *ASB1* (Ahmed et al., 2018). The jasmonate pathway, which includes transcription factors of great importance for regulating plant defenses and defence-related proteins, seems to be targeted by this effector. As a result, gene expression in response to pathogen infection is altered, increasing pathogen virulence.

### Alternative splicing as a regulator of interactions between plants and pathogens

Pre-messenger RNA (Pre-mRNA) splicing and alternative splicing (AS) are important mechanisms regulating gene expression. AS results in more than one mRNA, giving rise to proteins that may have different functions (Reddy et al., 2013; Staiger and Brown, 2013). It has been shown to play a role in plant adaptation to the environment and responses to biotic stress (Rigo et al., 2019). Splicing and AS are performed by spliceosomes. Like ribosomes, spliceosomes are complexes of small nuclear ribonucleoprotein particles (snRNPs) that are assembled and processed in the nucleolus (Patel and Bellini, 2008). AS has recently been implicated in plant immunity (Kufel et al., 2022) and pathogen effectors have been shown to modulate this process to hijack host-plant physiology to facilitate disease development. For example, a core component of spliceosomal snRNPs, SmD1, is targeted by root-knot nematode EFF18s in members of the Solanaceae and *Arabidopsis*, impeding its function in AS regulation and modulating the cell transcriptome to promote giant cell formation and nematode development (**Figure 1A**; Mejias et al., 2021, 2022). Nine of these splicing-regulatory effectors (SREs) were identified among 87 P*. infestans* effectors with an elegant splicing reporter system (Huang et al., 2020). Three SREs were shown to bind physically to known splicing factors, such as SR45, SR34 or the U1 snRNP 70K, which are known to be present in the nucleolus of *Arabidopsis thaliana* (Palm et al., 2016; Huang et al., 2020). These examples show that AS can be manipulated by pathogens with different infection strategies, to reprogram the host transcriptome in the interests of the pathogen.

### Nucleolar protein delocalisation/relocalization

In addition to interacting directly with various proteins in the nucleolus, some effectors also affect protein localization by preventing proteins from reaching the nucleolus or by trapping them within or outside the nucleolus, e.g. transcription factors, thereby disturbing nucleolar functions, e.g. the *Vibrio parahoemolyticus* VgpA (Hu et al., 2021), *C. burnetii* NopA and *B. abordus* NyxA and NyxB effectors (described above). Plant pathogens also use this strategy. The tobacco rattle virus produces the 16K protein, which interacts with COILIN, relocating this protein to the nucleolus, resulting in an activation of SA-dependent defense pathways and enhancing viral infection (Shaw et al., 2019). An RxLR effector of *P. infestans* causes the delocalization of the Pi04314 target protein from the nucleolus to the nucleoplasm, promoting late blight disease (Lee et al., 2023). The *A. euteiches* AeSSP1256 effector relocates the *Medicago truncatula* RNA HELICASE MtRH10 to the perinucleolar space and hinders its binding to plant RNA. MtRH10 is associated with ribosome-related genes, root development and defense (Camborde et al., 2022). Protein sequestration can disrupt diverse response pathways, providing pathogens with an opportunity to evade plant defenses. This process therefore constitutes an interesting target for pathogen effectors.

## Discussion

The effectors targeting nucleolar functions are still largely overlooked, despite demonstrations of the importance of controlling nucleolar processes for many animal and plant pathogens (Bierne, 2013; Kalinina et al., 2018; Iarovaia et al., 2021). Indeed, the nucleolus is the site of various key functions that can be exploited by pathogens (**Figure 1B**). Effectors targeting ribosome biogenesis are of particular interest. Ribosomes have been described as invariant protein complexes, but their composition has been shown to change at different life stages or under abiotic and biotic conditions, to meet cellular needs (Martinez-Seidel et al., 2020). This phenomenon has been observed in human cancer cells in particular (Penzo et al., 2019; Nait Slimane et al., 2020). As these changes in ribosome composition lead to differences in the proteome, it is not surprising that ribosome biogenesis is hijacked by various pathogens to exploit protein synthesis to their own advantage or to evade plant defenses. Boosting rRNA biogenesis could lead to an increase in the number of ribosomes, which could in turn be used to produce pathogen proteins (Vijayapalani et al., 2018). Pathogens that form specialized structures for invasion or reprogram plant-cell functions to their own advantage may also specifically target these cellular functions, as has been shown for nematodes during feeding-site establishment. Furthermore, ribosomes play a major role in the response to abiotic stresses and are currently being evaluated as a potential target for plant breeding (Dias-Fields and Adamala, 2022). Hijacking of the mediator complex or the splicing machinery also appears of particular interest to many pathogens, particularly as a means of disrupting defense gene expression. Initial studies on crops of agronomic interest, such as tomato, showed that the targeting of conserved spliceosomal proteins can lead to the development of broad-spectrum resistance through a loss of susceptibility to plant-parasitic nematodes (Mejias et al., 2022). Finally, the exclusion or retention of proteins in the nucleolus can significantly affect gene expression, enabling pathogens to evade plant defenses. Several studies have demonstrated the retention/sequestration of non-nucleolar proteins in the nucleolus during stress due to animal pathogens (Wang et al., 2019a). Access to the nucleolus therefore appears to be key for evading plant defenses and hijacking ribosome biogenesis. Given the many additional functions of the nucleolus, it would not be surprising to discover nucleolar effectors targeting other functions, such as genome reorganization or epigenetic modifications, which are attracting increasing interest in the domain of plant pathology. However, despite the discovery of new nucleolar functions, the targeting of these functions by effectors remains insufficiently explored.

Analyses to date of the cellular localization of predicted plant-pathogen effectors, with or without searches for specific localization signals, such as NLS (nuclear localization signal) or NoLS (nucleolar localization signal), have led to the identification of effectors that localize in the plant nucleolus but are also frequently present in the nucleoplasm. Future studies should adopt a more systematic approach, searching for effectors with localization signals and taking into account all the key functions of the nucleolus. Such approaches are possible with bioinformatic tools, such as Prediction of Protein Sorting Signals and Localization Sites in Amino Acid Sequences (PSORT; Nakai and Horton, 1999), which can identify such localization signals in addition to NoD, which specifically predicts NoLS (Scott et al., 2011). Proteins may also enter the nucleolus by binding to nucleolar proteins, such as FIB, and this may provide effectors with an alternative way of aiming the nucleolus. Moreover, the molecular targets of many effectors reported to target the nucleolus have yet to be characterized (Wang et al., 2019b; Chen et al., 2020). In-depth functional studies are required to decipher the underlying mechanisms and shed light on the multiple roles of the nucleolus and the ways in which pathogens hijack these key functions, to facilitate the development of better plant protection strategies.

Given the diverse functions of the nucleolus, effectors manipulating this nuclear organelle should not be ignored, as approaches focusing on these effectors could make a major contribution to efforts to move from qualitative resistance to quantitative resistance, and to the establishment of durable resistance in the field.

## Author contributions

SR-R: Writing – original draft. FP: Writing – review & editing. MQ: Funding acquisition, Supervision, Writing – review & editing. BF: Funding acquisition, Supervision, Writing – review & editing.
